# Why does moral reasoning not improve in medical students?

**DOI:** 10.5116/ijme.55d4.c8e4

**Published:** 2015-08-27

**Authors:** Sebastian Sheehan, Andrew Robbins, Thomas Porter, John Manley

**Affiliations:** School of Medicine, Cardiff University, Cardiff, Wales, United Kingdom

**Keywords:** moral reasoning, medical students

## Introduction

Murrell’s article discussing the failure in development in moral reasoning at medical school addresses an issue of great concern.^1^ It is of paramount importance for doctors to be capable of sophisticated ethical and moral reasoning, as this promotes a consideration of patients on an individual level and the adoption of a holistic approach to their care. The resultant concordance of doctor and patient may increase the likelihood of mutually satisfactory decisions, patient compliance and beneficial clinical outcomes.

The finding that medical students do not seem to progress their moral reasoning is concerning, and outlined herein are some potential factors responsible for this arrest in development of metacognition. Following this a tentative solution to the problem is offered.

### Education by guidelines

One primary aim of any medical school is to produce doctors that are safe and effective in their junior jobs, able to approach acute emergencies whilst diagnosing and prescribing safely. This is achieved through knowledge of preordained drug doses, written guidelines and diagnostic manuals, which are effective in disseminating important information.^2^ Teaching of protocol-based care also continues after medical school, featuring prominently in the UK foundation programme.^3^ However as alluded to in Murrell’s article, it remains unclear whether such a rule-based, factual approach facilitates the higher philosophical thought key to moral and metacognitive development.^1^ As suggested in the literature, the increasing focus on adherence to guidelines reduces scope for consideration of ethical values and for moral reasoning when making clinical decisions.^4^ Consequently adherence to such rules during medical school may limit the higher thought needed to develop moral reasoning to the post-conventional stage.

### Negative emotional experiences from ‘informal’ moral learning

Ethical and moral education is an established part of the medical school curriculum, delivered concomitantly with professionalism. Although students feel that formal ethical education is important and relevant, it is inadequate to facilitate development of moral reasoning.^1^^, ^^5^ The informal curriculum of the clinical environment, with its cynicism, dark humor and sometime unethical behaviour might act antagonistically to formally delivered content, so arresting development of moral reasoning skills. It has been observed that students feel inexperienced and uncomfortable around the most unwell patients and their families – the very situations that often provide invaluable emotional, moral and ethical experience.^6^ The unreliable nature of the informal curriculum may fail to nurture students as needed in these situations, negating net metacognitive benefit throughout medical school.

### Insufficient emotional investment in patients

There is no substitute for patient contact when developing clinical reasoning. Students gain experience through interaction with different patients in a variety of clinical settings.^7^^, ^^8^ However a wide range of learning objectives must be met during relatively short clinical attachments, potentially limiting the scope for emotional investment in individual patients. As such student-patient interactions tend to be brief and superficial, restricting appreciation of the patient’s life and reducing emotional exposure to disease and treatment processes. Therefore students are not prompted to dwell on one moral issue, mulling over complexities and different philosophical approaches. We feel that this might explain why doctors who care for their patients over weeks and months show more developed moral judgment than their students.^1^

Medical students rotate regularly through clinical attachments in different specialties, meeting new supervising clinicians who must be satisfied to allow progression through the course. The professionalism of the student is not often measured by emotional investment in a patient or participation in their care, but by dress, attendance and meeting set learning goals.^9^ This can provide a significant source of stress in clinical students, who become preoccupied with getting clinical skills or outcomes 'ticked off a list', further distracting them from moral and metacognitive development.^10^

### How to improve moral reasoning?

The curricular integration of regular small group teaching sessions would be an excellent way to facilitate moral development, and act as a counterbalance to any negative effects of the ‘informal curriculum’.^6^ For ethical and moral subject matter intellectual engagement is key and so exploratory group discussion is of paramount importance as it facilitates debate; thus maximizing involvment.^11^^,^^12^ As they approach the end of the course senior students could fulfill the role of facilitator, delving deeper into ethics and morals.

Literature and film might facilitate moral education as suggested by Self et al.^13^ An optional reading or watching list alongside a crib sheet guiding moral reasoning might be beneficial, enhancing the perceived relevance of subsequent teaching sessions. A recent study evaluated the effect of professionally representative television on moral reasoning in medical students. Their findings and earlier work by Goodman support the suggestion that these informal methods can boost the development of moral reasoning and have a unique pedagogical value.^14^^,^^15^ Crucially, this relaxed approach might encourage students to be more emotionally open to the subject matter, whilst not adding to workload-related stress. This is a relatively novel way of educating medical students therefore further evaluation is required. 

In conclusion the reasons for arrested development of moral reasoning in medical students are varied and multifactorial. Some will prove difficult to eliminate as they may be inherent to the course. However it should be simple to supplement a medical curriculum with teaching that nurtures these skills. It is of paramount importance for thepatients and doctors of tomorrow that we do so.

### Conflict of Interest

The authors declare that there are no conflicts of interest.
